# The complete chloroplast genome and phylogenetic analysis of *Pterospermum heterophyllum* (Malvaceae)

**DOI:** 10.1080/23802359.2026.2619279

**Published:** 2026-01-24

**Authors:** Yihong Luo, Yang Ni, Jiahua Chen, Guoan Shen, Lichai Yuan

**Affiliations:** a Institute of Medicinal Plant Development, Chinese Academy of Medical Sciences & Peking Union Medical College, Beijing, PR China; b School of Chemistry and Chemical Engineering, Guangdong Pharmaceutical University, Guangzhou, Guangdong Province, PR China

**Keywords:** Yao medicine, medicinal plant, ethnomedicine, plastid, *Pterospermum heterophyllum*

## Abstract

*Pterospermum heterophyllum* Hance (1868), a member of the Malvaceae family, is widely distributed across southwestern and southeastern China and has been traditionally used in folk medicine to treat rheumatoid arthritis and inflammation-related diseases. The circular genome is 162,857 bp long and exhibits a typical quadripartite structure, consisting of an LSC (91,356 bp), SSC (20,567 bp), and two IRs (25,467 bp each). A total of 130 genes were annotated. The multiple sequence alignment revealed that infA gene contained a large 3′ deletion truncating the coding sequence. Phylogenetic analysis revealed that *P. heterophyllum* is closely related to *P. kingtungense* and *P. truncatolobatum*.

## Introduction

*Pterospermum heterophyllum* Hance (1868), also known as *Pterospermum levinei* Merr. (Hu 1924) is a perennial plant in the family Malvaceae. It is widely distributed across the southwestern, southeastern, and southern regions of China. The root of *P. heterophyllum* has traditionally been used in folk medicine to treat rheumatoid arthritis (RA) and other inflammation-related diseases. A total of 73 chemically active compounds have been identified from it, including 34 flavonoids. Animal experiments were also conducted to test the pharmacological effects of *P. heterophyllum*. In an arthritis rat model, oral administration of *P. heterophyllum* extract for 22 days significantly ameliorated the knee joint damage and decreased the spleen and thymus indices (Yang et al. 2020). It also contained triterpenoids with antitumor activity (such as betulinic acid), which can inhibit lung cancer cells (A549) (Li et al. 2009). Extracts from its leaves and stems also exhibit antibacterial and antifungal activities, showing inhibitory effects against Gram-negative bacteria and fungi, indicating their potential for treating infectious diseases (Tu et al. 2024).

The chloroplast, a crucial organelle for photosynthesis in plants, contains a genome (cpDNA) that demonstrates a conserved structure, maternal inheritance, and a moderate mutation rate (Daniell et al. 2016; De Las Rivas et al. 2002). These attributes render it a significant molecular marker for examining plant phylogeny, speciation, and adaptive evolution (Li et al. 2015; Smith 2017; Jansen et al. 2007). The chloroplast genome contained several coding and non-coding regions, with sequence variants providing high-resolution molecular evidence for taxonomic classification and genetic distinction among closely related species (Dobrogojski et al. 2020). In recent years, comparative analyses utilizing entire chloroplast genomes have emerged as powerful methods for clarifying intricate taxonomic controversies and reconstructing deep phylogenetic relationships (Chong et al. 2022).

As the foundational botanical source of *P. heterophyllum*, the complete sequencing of its chloroplast genome had not yet been reported. To gain a deeper understanding of and harness the therapeutic potential of *P. heterophyllum*, and to ensure its safety in clinical applications, a comprehensive investigation of its chloroplast genome is of paramount importance, as numerous molecular markers can be identified from the chloroplast genome for the differentiation of varieties of *P. heterophyllum*.

This study aims to sequence, assemble, and annotate the whole chloroplast genome of *P. heterophyllum*. The results can be used to identify markers for precise identification of the resources of this species. Furthermore, the phylogenetic analysis based on the chloroplast genome data could improve understanding of the phylogenetic relationships within other species of the the *Pterospermum* genus and the Malvaceae family.

## Materials and methods

**Figure 1.**
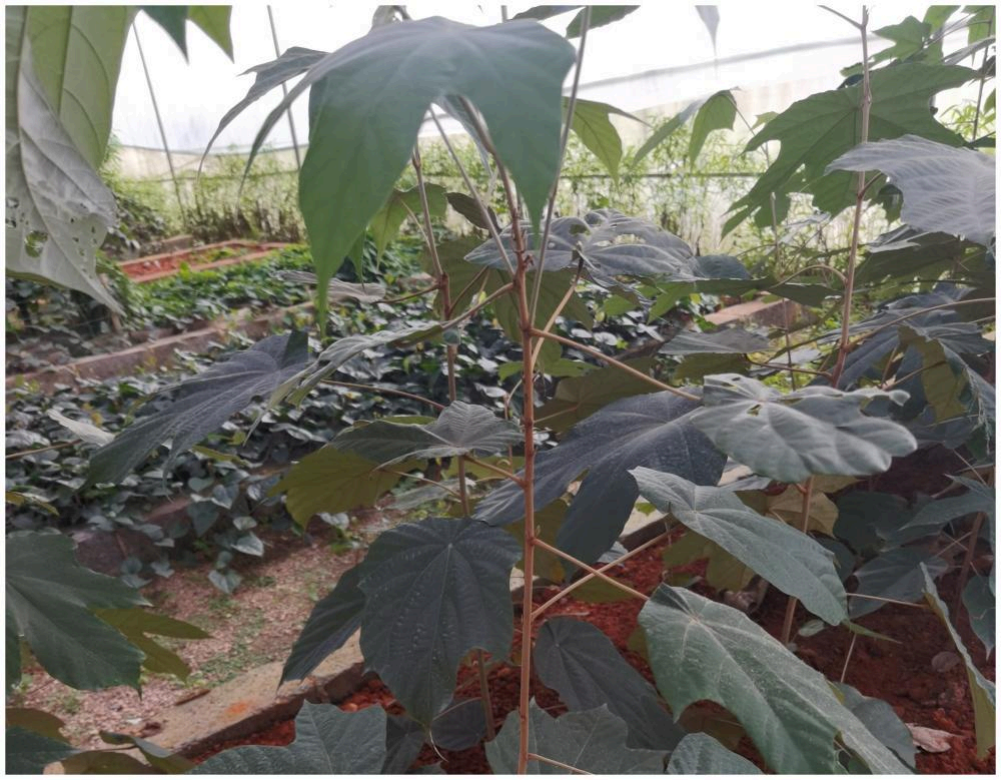
Leaf of *P. heterophyllum*, photographed by Jiahua Chen in Jinxiu Yao Autonomous County, Guangxi, China (24.177468**°** N, 110.008461**°** E). The leaf blade is oblong to ovate-oblong, 7–15 cm long, with an obtuse, truncate, or obliquely cordate base. The adaxial surface is sparsely pubescent, whereas the abaxial surface is covered with a dense yellowish-brown tomentum. The species is heterophyllous: juvenile leaves (and leaves on growing shoots or buds) are often deeply palmately lobed with the petiole inserted near the leaf center (peltate), while mature leaves are entire and have the petiole attached at the leaf margin.

Young and healthy leaves of *P. heterophyllum* were collected from Jinxiu Yao Autonomous County, Guangxi Province, China (24°10′38.88″N, 110°0′30.46″E) (Figure 1). The taxonomy ID is 190904. The plant specimen was identified by Zhaosheng Pang, and a voucher specimen (JXHC091) was deposited at the Institute of Medicinal Plant Development, Chinese Academy of Medical Sciences & Peking Union Medical College (Contact: Guoan Shen, gashen@implad.ac.cn).

Total genomic DNA was extracted using a modified CTAB method. High-quality DNA was then sequenced on the Illumina HiSeq 2500 platform (Illumina, San Diego, CA, USA) by Wuhan GrandOmics Biotechnology Co., Ltd.

To obtain clean sequencing reads, we used FASTP v0.24.1 (Chen et al. 2018) to perform quality control on the raw sequencing reads, filtering with a quality score threshold of Q15. The clean sequencing reads were assembled into a complete chloroplast genome using GetOrganelle v1.7.7.1 (Jin et al. 2020) with default parameters. The resulting circular genome was visualized using Bandage v0.8.1 (Jin et al. 2020). BWA v0.7.17 (Li et al. 2009) was selected for read mapping due to its proven accuracy and efficiency in handling chloroplast genomic data. Chloroplast genomes are small, conserved, and circular, requiring precise alignment of Illumina short reads for accurate coverage calculation. While other aligners like Bowtie2 are also effective, BWA’s algorithm is particularly robust and has been extensively used in numerous published chloroplast genomics studies. The subsequent use of Samtools v1.13 (Danecek et al. 2021), which integrated seamlessly with BWA’s output format, represented a standardized and efficient pipeline for generating the coverage statistics. Genome annotation was performed by using CPGAVAS2 (Shi et al. 2019), with *P. kingtungense* (NC_042885) as the reference; all other parameters were set to the default values on the CPGAVAS2 webserver (Shi et al. 2019). Manual corrections of the annotation of tRNA genes, start and stop codons, and intron/exon boundaries were performed using Apollo (Dunn et al. 2019). The chloroplast genome map, the cis-splicing genes, and the trans-splicing gene were generated using CPGView (Liu et al. 2023).

For phylogenetic analysis, we retrieved 18 complete chloroplast genomes from GenBank belonging to the order Malvales. The ingroup consisted of 16 species from the family Malvaceae, selected as the top 16 closest relatives based on GenBank’s BLAST results, while the outgroup comprised two randomly chosen species from a different family (Thymelaeaceae) of the same order (Malvales). By using PhyloSuite (Xiang et al. 2023), 66 shared protein sequences were extracted from all species for downstream analysis. Two species, *Aquilaria yunnanensis* (NC_036940) and *Aquilaria crassna* (MN125348), were used as outgroups. Multiple sequence alignments were generated utilizing MAFFT v7.505 (Katoh et al. 2002). Maximum likelihood (ML) trees were constructed using IQ-TREE v2.2.0 (Lanfear et al. 2020) under the model cpREV (General Reversible Chloroplast), which was selected because it accounted for the unique evolutionary constraints of chloroplast-encoded protein sequences. Node support was assessed with 1000 bootstrap replicates. The resulting phylogenetic tree was visualized using the Interactive Tree of Life (iTOL) online tool (https://itol.embl.de/) (Letunic et al. 2024).

We found that the *infA* gene in *P. heterophyllum* had premature termination. To confirm this observation, we verified the assembly accuracy of the *P. heterophyllum infA* coding sequence and its flanking 50 bp regions. As shown in Figure S4, the sequencing depth across this entire region exceeded 600×, confirming that the assembly of the *infA* locus was reliable.

To confirm that the *infA* gene is indeed truncated, we performed a comparative analysis using a set of plastome references. We downloaded the open-source CPGAVAS2 Singularity container, an instruction can be found at (http://www.1kmpg.cn/cpgavas2c/README.pdf), from which we extracted the complete chloroplast reference genome database comprising 2,544 curated plastomes. From these plastomes, the *infA* gene was retrieved from each genome and aligned with the *P. heterophyllum infA* sequence. Using a significance threshold of evalue ≤ 0.05, we identified 50 homologous *infA* gene sequences with statistically supported similarity to *P. heterophyllum* (Table S4). Among these, 31 sequences with pairwise identity greater than 80% and bitscore greater than 100 were selected for visualization in Jalview v2.11.5.0 (Waterhouse et al. 2009).

## Results

The raw sequencing reads have been deposited in the GenBank Sequence Read Archive (SRA) under accession number SRR33807224. They comprised 150 bp paired-end reads with about 15.2 G of raw data and 14.9 G of clean data. The complete chloroplast genome sequence of *P. heterophyllum* was submitted to the GenBank database under accession number PV738961. The assembly exhibited high quality, with an average depth of coverage of 1001.65x with no uncovered regions (Figure S1).

**Figure 2.**
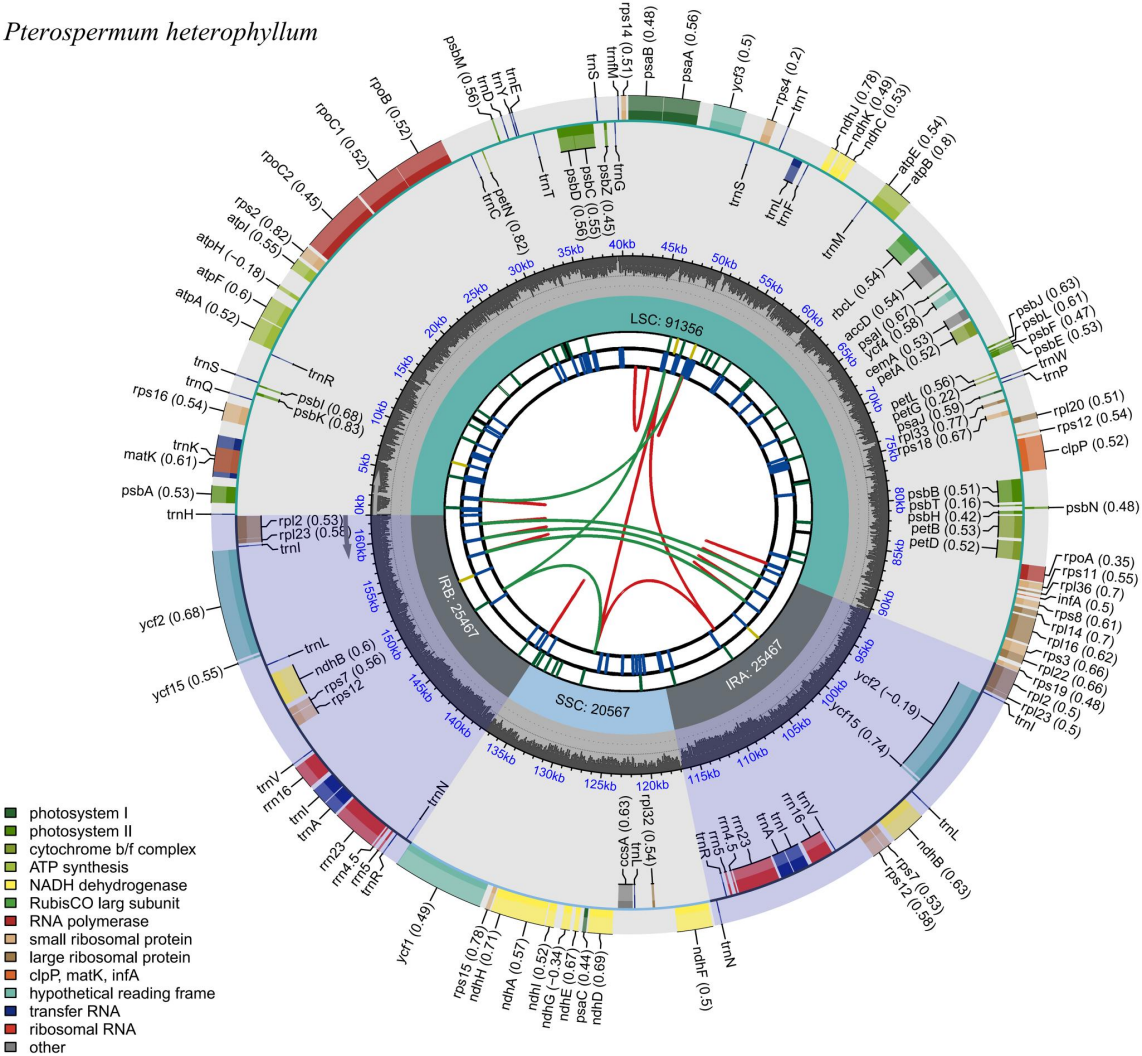
Schematic map illustrating the overall features of the chloroplast genome of *P. heterophyllum*. The map comprises six concentric tracks by default. From the center outward: the first track displays dispersed repeats; the second shows long tandem repeats as short blue bars; the third indicates short tandem repeats (microsatellites) as colored bars. The fourth track marks the structural regions of the genome, including SSC, LSC, inverted repeats A (IRA) and B (IRB). The fifth track plots the GC content across the genome. The sixth track presents annotated genes, with optional codon usage bias shown in parentheses after gene names. Genes are color-coded based on functional categories, as indicated in the legend at the bottom left. Genes transcribed on the inner and outer circles are oriented clockwise and counterclockwise, respectively.

The chloroplast genome of *P. heterophyllum* was 162,857 bp in length and had an overall GC content of 36.43%. It exhibited the typical quadripartite structure, consisting of a large single-copy (LSC) region of 91,356 bp, a small single-copy (SSC) region of 20,567 bp, and a pair of inverted repeats (IR) regions, each had a size of 25,467 bp (Figure 2). The GC content varied across different regions: 34.02% in the LSC, 30.85% in the SSC, and 43.00% in the IRs. A total of 130 genes (112 unique genes) were annotated in the chloroplast genome, including 87 protein-coding genes (80 unique), 35 transfer RNA (tRNA) genes (28 unique), and 8 ribosomal RNA (rRNA) genes (4 unique). Among the protein-coding genes, 11 were identified as cis-splicing genes and contained two exons (Figure S2). Two genes: *ycf*3, *clp*P, were also cis-splicing genes and each contained three exons. The *rps*12 gene was characterized as a trans-splicing gene (Figure S3). It contains three exons; two of the exons (exon2 and exon3) were duplicated. We showed the number of genes in Table S1, and showed the gene-by-gene annotation information in Table S2.

**Figure 3.**
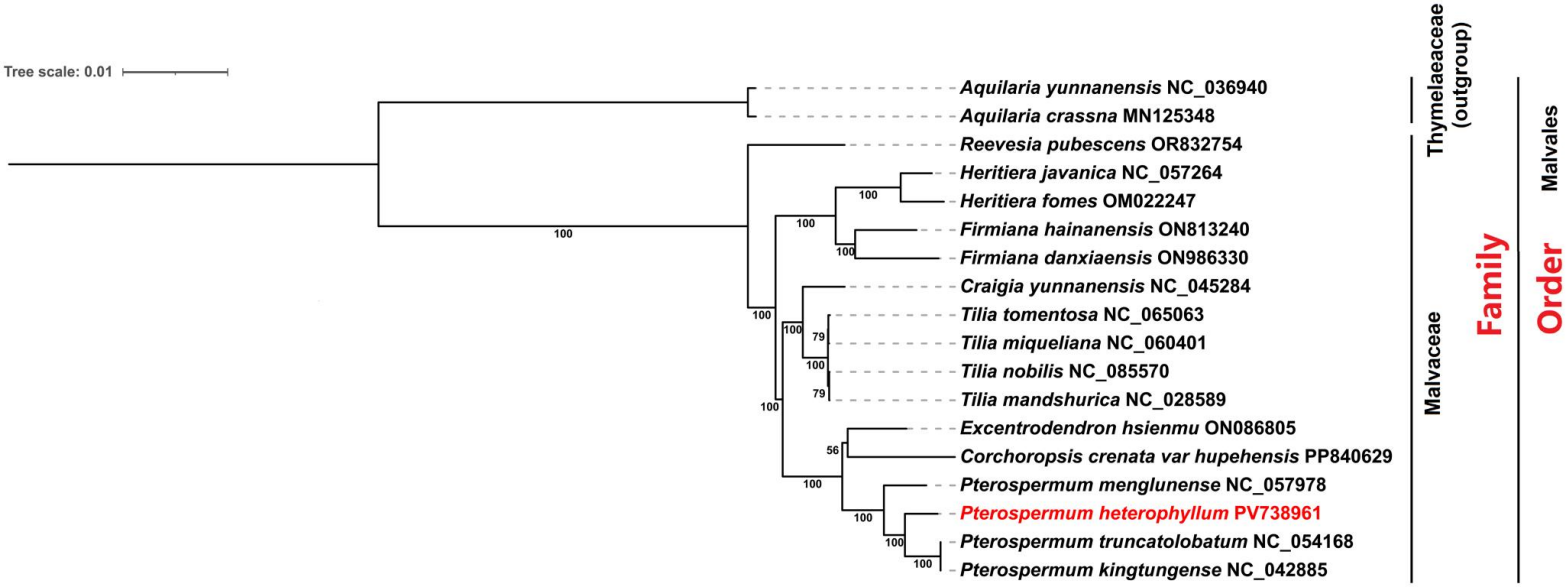
Phylogenetic tree of 16 additional malvaceae species based on complete chloroplast genomes, inferred using the ML method from 66 shared protein sequences. Maximum-likelihood phylogeny of selected malvales taxa inferred from molecular sequence data. Species names are followed by GenBank accession numbers; bootstrap support values (percent) are shown at nodes. Branch lengths are proportional to genetic change. Thymelaeaceae was used as the outgroup (family and order labels are shown to the right). The *P. heterophyllum* (PV738961) is highlighted in red. The sequences used for constructing the phylogenetic tree are as follows. *Heritiera javanica* (Yoocha T, et al. 2023), *H. fomes* (Yoocha T, et al. 2023), *Firmiana hainanensis* (Tan F, et al. 2023), *F. danxiaensis* (Chen SF, et al. 2024), *Craigia yunnanensis* (Wariss HM, et al. 2019), *Tilia mandshurica* (Cai J, et al. 2015), *Corchoropsis crenata* (Jung J, et al. 2024), *Pterospermum menglunense* (Guan-Song Y, et al. 2021), *P. heterophyllum* (this study)*, P. truncatolobatum* (Wang J-H, et al. 2021), *P. kingtungense* (Wang Z, et al. 2018). The following sequences are available on GenBank but remain unpublished: *Reevesia pubescens* (OR832754), *Tilia tomentosa* (NC_065063), *T. miqueliana* (NC_060401), *T. nobilis* (NC_085570) and *Excentrodendron hsienmu* (ON086805). *Aquilaria yunnanensis* (Hishamuddin MS, et al. 2020) and *A. crassna* (Hishamuddin MS, et al. 2020) were selected as the outgroup species.

Phylogenetic analysis was conducted based on 66 shared protein sequences from 18 species across nine genera within the Malvales order, including two species from the Thymelaeaceae family as the outgroup. Table S3 lists all taxa used in phylogenetic analyses with full accession numbers. The results indicated that *P. heterophyllum* is closely related to *P. truncatolobatum* and *P. kingtungense* with strong bootstrap support (Figure 3).

The resulting multiple sequence alignment demonstrated that, while most angiosperms contained a conserved and complete open reading frame for *infA*, the *P. heterophyllum* sequence exhibited a large deletion in the 3′ end of the coding sequence (Figure S5). This deletion truncates the coding sequence and strongly indicated that the chloroplast *infA* gene in *P. heterophyllum* was likely nonfunctional.

## Discussion and conclusion

In the present study, we obtained the chloroplast genome of *P. heterophyllum.* It was highly similar to those of other genera within the Malvaceae family, particularly regarding the overall gene structure and composition. The GC contents of Malvaceae chloroplast genomes ranged from 36.4% and 37.2%, and their length varied from 158.6 kb to 163.5 kb (Yang et al. 2018; Wu et al. 2023; Wariss et al. 2019).

Previous studies had shown that the *infA* genes in multiple genera of the Malvaceae family were likely pseudogenes. However, the *infA* genes in the closely related genus *Eriolaena* were found to be complete. This indicates that the phenomenon is not a shared characteristic of this evolutionary branch but occurred independently (Jung et al. 2024). By using DNA gel electrophoresis and hybridization, the chloroplast *infA* gene may have undergone at least 16 independent loss events in angiosperms outside the rosids clade (Millen et al. 2001). As a result, we propose that the loss of *infA* in *P. heterophyllum* likely represented an independent evolutionary event.

Phylogenetic analysis showed that *P. heterophyllum* had a close relationship with *P. truncatolobatum* and *P. kingtungense.* This is consistent with the conclusion of a previous study (Jung J, et al. 2024). Among the *Pterospermum* species sampled, *P. heterophyllum* and *P. heterophyllum* had the closest sister species relationship.

In summary, the complete chloroplast genome of *P. heterophyllum* has been assembled and is reported for the first time. The results provided a valuable resource for species classification and phylogenetic studies within the *Pterospermum* genus and the Malvaceae family. Further studies with broader taxon sampling and multi-genome data will help deepen our understanding of the evolutionary history of *Pterospermum* species.

## Supplementary Material

Supplemental Material

## Data Availability

The genome sequence data that support the findings of this study are publicly available from the GenBank database under accession number PV738961. The associated BioProject, BioSample, and SRA accession numbers are PRJNA1271244, SAMN48857971, and SRR33807224.

## Species conservation statement

We confirm full compliance with IUCN policies, the Convention on Biological Diversity, and CITES. *P. heterophyllum* has been listed as Near Threatened in China’s Biodiversity Red List—Volume of Higher Plants (2020). However, it is not listed as a nationally protected plant in the collection location, and it has not been included in the IUCN Red List of Threatened Species.
