# Supplementary material for: The complete chloroplast genome and phylogenetic analysis of Pterospermum heterophyllum (Malvaceae)
Source: Mitochondrial DNA B Resour. 2026 Jan 24;11(2):295–300. doi: 10.1080/23802359.2026.2619279 (PMC12833906; doi:10.1080/23802359.2026.2619279)

**Figure S1**. Overall coverage and sequencing depth of the *P. heterophyllum* chloroplast genome assembly. The X-axis represents genome position, and the Y-axis shows sequencing depth at each base.


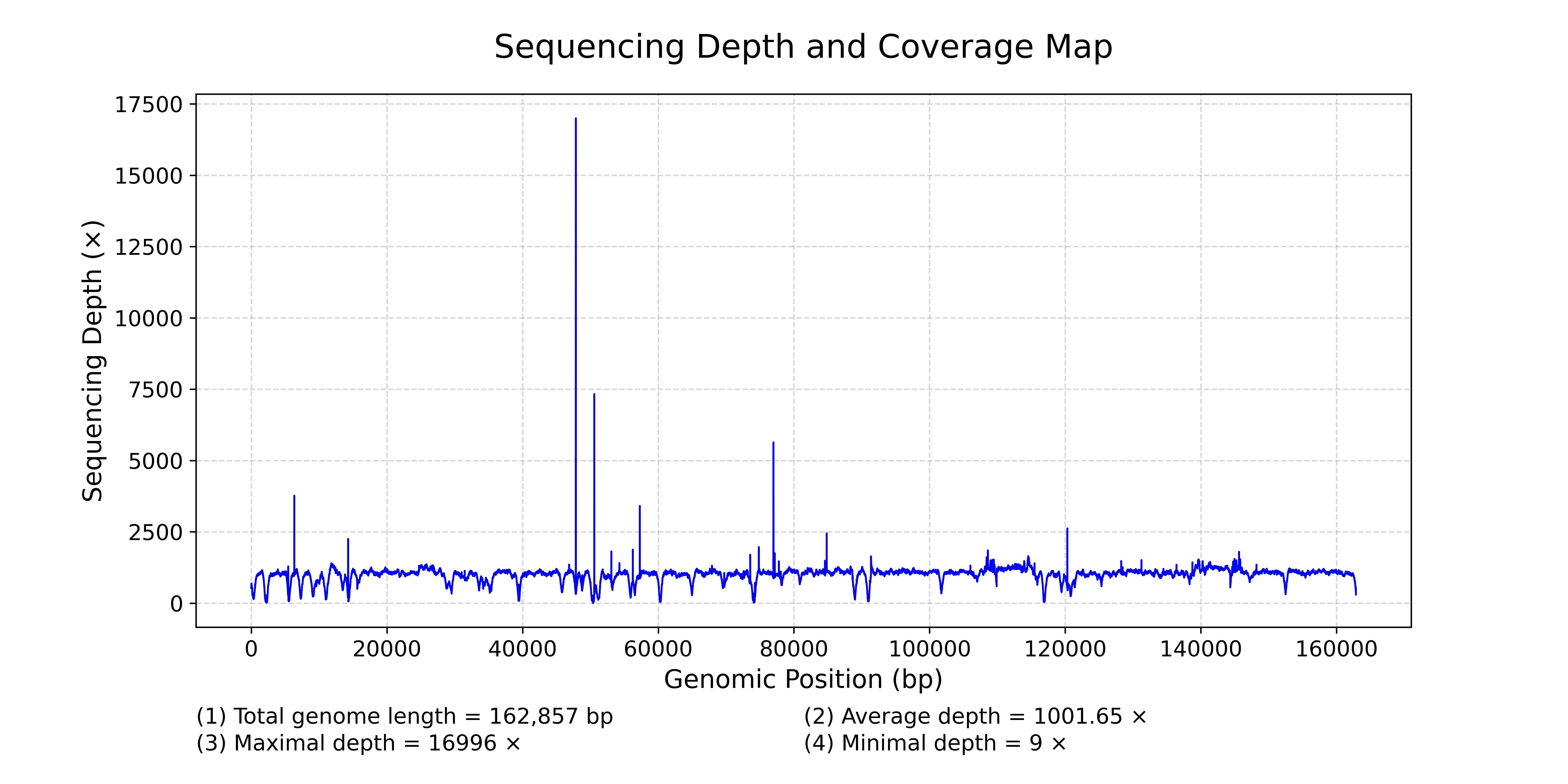


**Figure S2.** Schematic map of cis-spliced genes in the *P. heterophyllum* chloroplast genome. Genes are arranged vertically from top to bottom according to their positions in the chloroplast genome. Gene names are shown on the left, with gene structures depicted on the right. Exons are shown in black, introns in white, and arrows indicate the direction of gene transcription (sense strand). Exon and intron lengths are not drawn to scale and serve only to illustrate the gene structure.


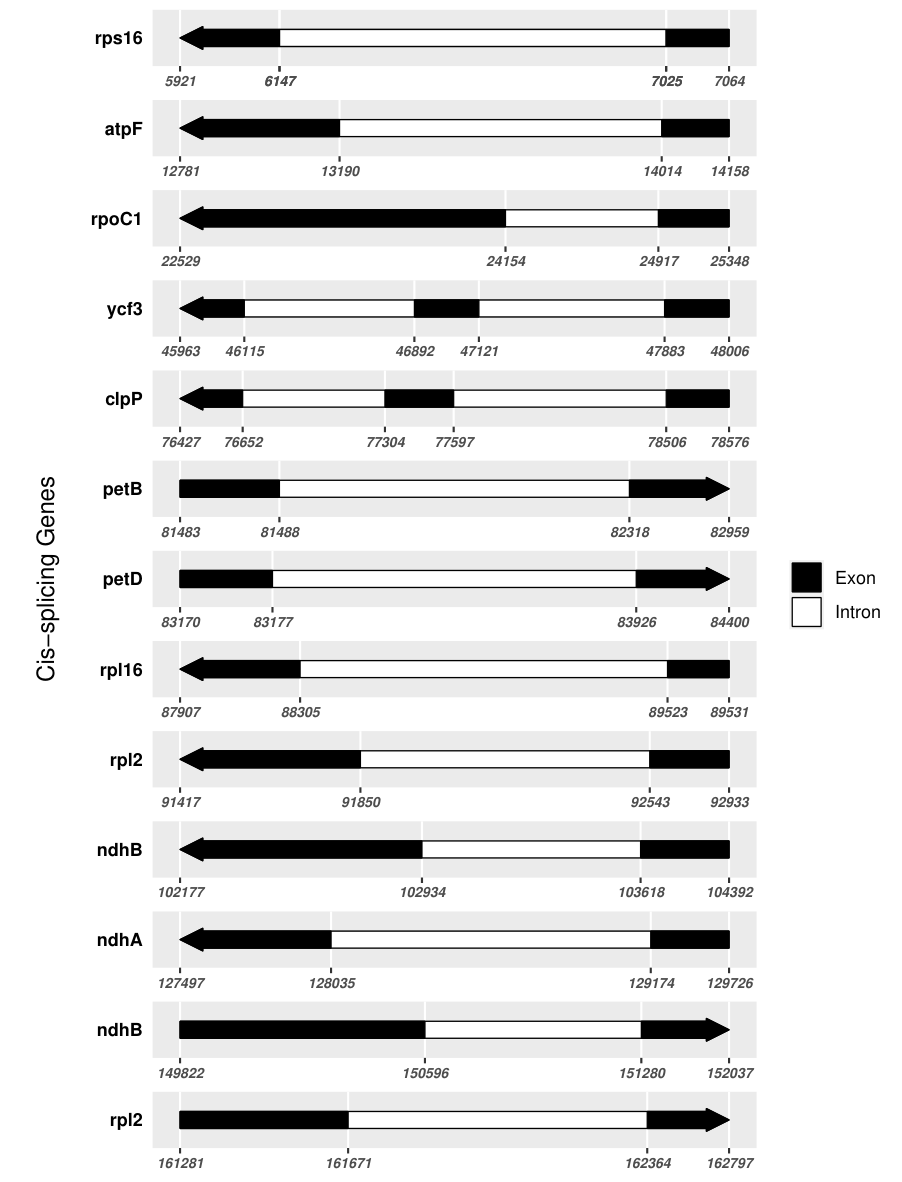


**Figure S3.** Schematic map of the trans-spliced gene *rps*12 in the *P. heterophyllum* chloroplast genome. The gene consists of three unique exons, two of which are loaded within the IR regions.


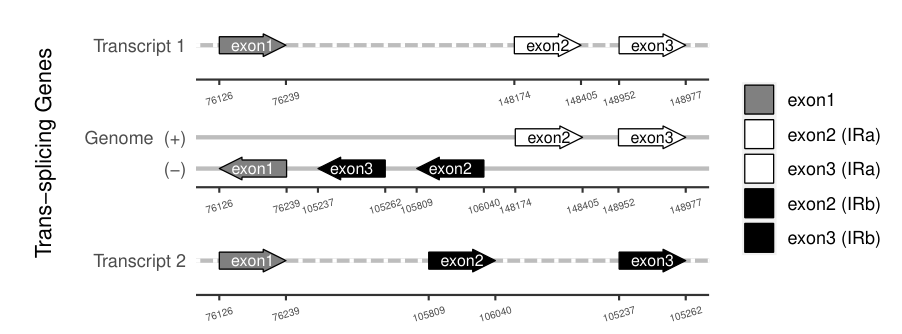


**Figure S4**. Overall coverage and sequencing depth of the *infA* gene in *P. heterophyllum* chloroplast genome assembly. The X-axis represents genome position, and the Y-axis shows sequencing depth at each base.


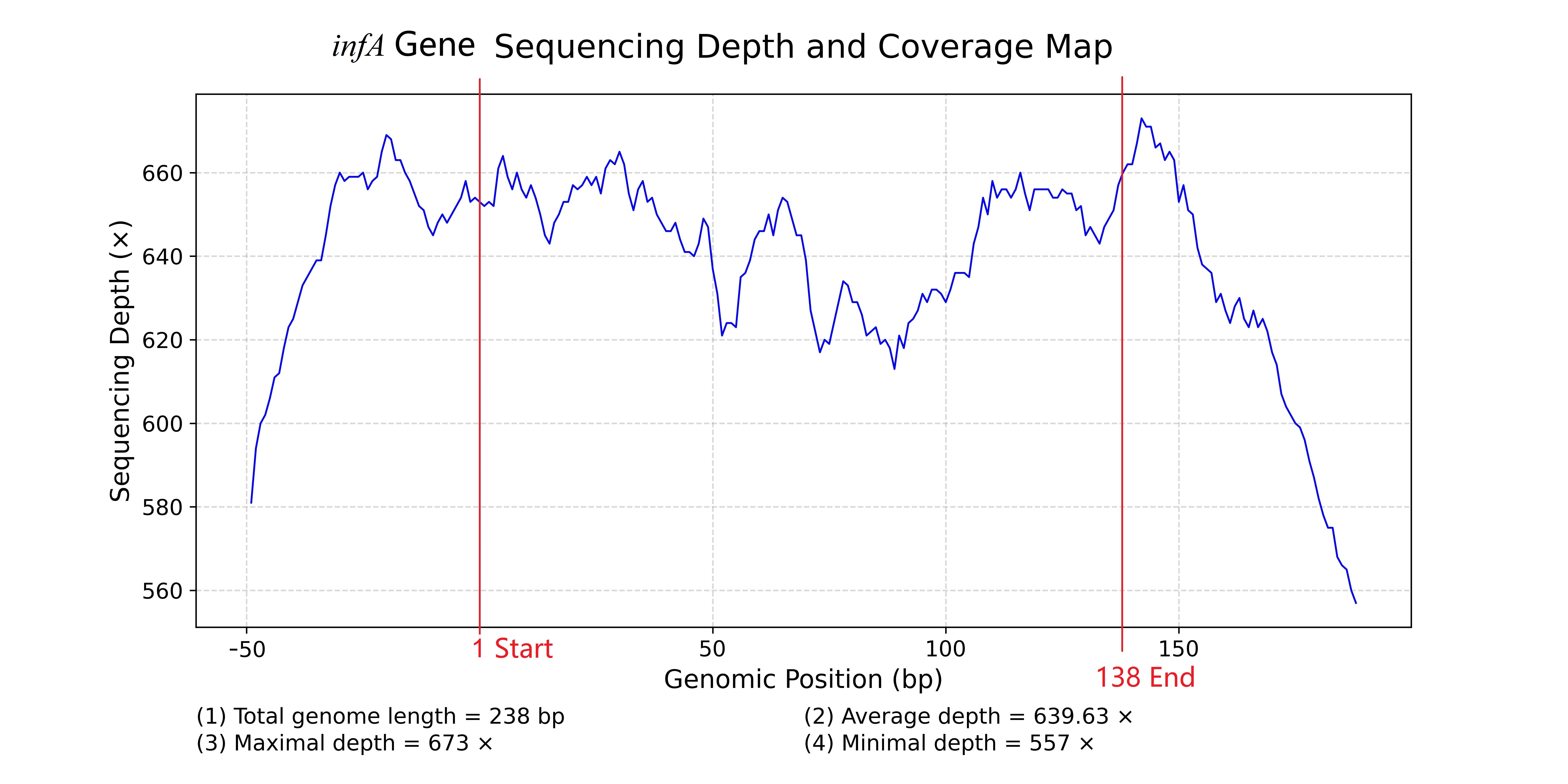


**Figure S5**. A comparative alignment of the *infA* gene nucleotide sequences from representative angiosperms is shown. Conserved positions across taxa are highlighted by shared color patterns, while variable sites appear in mixed colors. The *infA* gene of *P. heterophyllum* is highlighted in red.


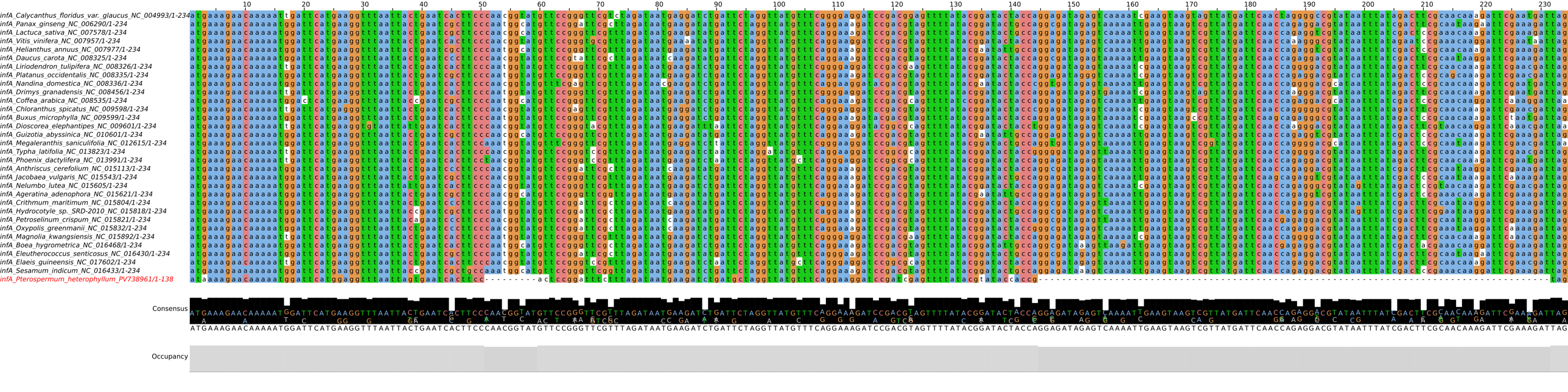

Supplement: Supplemental Material [file TMDN_A_2619279_SM3039.docx]
